# Interactive Multigrid Refinement for Deformable Image Registration

**DOI:** 10.1155/2013/532936

**Published:** 2013-10-22

**Authors:** Wu Zhou, Yaoqin Xie

**Affiliations:** Shenzhen Key Laboratory for Low-Cost Healthcare, Shenzhen Institutes of Advanced Technology, Chinese Academy of Sciences, Shenzhen 518055, China

## Abstract

Deformable image registration is the spatial mapping of corresponding locations between images and can be used for important applications in radiotherapy. Although numerous methods have attempted to register deformable medical images automatically, such as salient-feature-based registration (SFBR), free-form deformation (FFD), and demons, no automatic method for registration is perfect, and no generic automatic algorithm has shown to work properly for clinical applications due to the fact that the deformation field is often complex and cannot be estimated well by current automatic deformable registration methods. This paper focuses on how to revise registration results interactively for deformable image registration. We can manually revise the transformed image locally in a hierarchical multigrid manner to make the transformed image register well with the reference image. The proposed method is based on multilevel B-spline to interactively revise the deformable transformation in the overlapping region between the reference image and the transformed image. The resulting deformation controls the shape of the transformed image and produces a nice registration or improves the registration results of other registration methods. Experimental results in clinical medical images for adaptive radiotherapy demonstrated the effectiveness of the proposed method.

## 1. Introduction

Radiotherapy is an image-guided treatment, and imaging is involved in every key step of the process. The evolution of radiation therapy has been strongly correlated with the development of imaging techniques [1]. The term of image-guided radiation therapy (IGRT) is employed loosely to refer to newly emerging techniques of radiation planning, patient setup, and delivery procedures that integrate cutting-edge image-based tumor definition methods, patient positioning devices, and/or radiation delivery guiding tools. These techniques combine new imaging tools, which interface with the radiation delivery system through hardware or software, and state-of-the-art 3D conformal radiation therapy (CRT) or intensity modulated radiation therapy (IMRT) and allow physicians to optimize the accuracy and precision of the radiotherapy by adjusting the radiation beam based on the true position of the target tumor and critical organs [2]. This increased accuracy justifies a smaller clinical target volume to planning target volume (CTV-PTV) margin, thus decreasing the consequent collateral damage to the normal tissues. While IGRT is certainly a step forward for radiation oncology, the efficacy of these image-guided treatments depends on a treatment plan optimized using these images.

One of the key questions in image guidance is how the information is used to modify treatment. If the target and organs at risk (OARs) can be delineated on online volumetric images, it is possible to generate an adaptive treatment plan. Replanning theoretically provides the highest precision and does not need specialized hardware such as the robotic couch. However, online replanning requires superior online image quality, as well as fast and robust algorithms, to perform automatic region-of-interest (ROI) delineation, dose calculation, and beamlet weight optimization. Various methods are used clinically to increase the speed of ROI delineation, including atlas-based segmentation, ROI propagation, and deformable image registration [3]. Deformable image registration is a fundamental task in medical image processing due to its potential clinical impact [4]. For instance, the advantage of deformable image registration in adaptive radiotherapy is that the deformation field can be used for nonrigid dose accumulation [5].

The process of deformable image registration consists of establishing functional and/or spatial anatomical correspondences between different images. The term deformation is often used to denote the fact that the observed images are associated through a nonlinear dense transformation or spatially varying deformation model [6]. Deformable image registration has been studied in great detail, and numerous methods have attempted to register deformable medical images automatically, such as salient-feature-based registration (SFBR) [7, 8], free-form deformation (FFD) [9], and Demons [10, 11]. SFBR is a point-based registration approach which uses salient features that are prominent and distinctive features in the image. The features are extracted in two images using an interest point detector and are then matched for correspondence. The correspondent features are then used to interpolate a nonrigid transformation using the thin-plate-spline method [12]. In order to recover the local geometric differences well between anatomic structures by SFBR, it is also assumed that there are enough correspondent landmarks in local geometric differences areas. Typically, a large number of reliable corresponding anchor points are required for accurate registration [13]. However, it is not often fulfilled in clinical applications; for instance, in homogenous regions, the feature-based method may fail when few or no salient features locate in these corresponding regions. The Demons algorithm uses image intensity values and assumes that pixels presenting the same anatomical points on each image have the same intensity values, and thus it is appropriate for monomodality image registration. When the local geometric deformation is large or images are in multimodality, the Demons algorithm becomes difficult to handle. FFDs are one of the most common types of transformation models in medical images. The advantage of the transformation model lies in its simplicity, smoothness, and ability to describe local deformations with few degrees of freedom. However, misregistration in the difference image after such deformable registration is still viable. The main reason for this is the limited flexibility of deformation registration methods to describe complex local deformations. In addition, most of the existing methods based on energy minimization or optimization may fail in clinical settings due to the suboptimal solutions and excessive running time. Some recently proposed methods [14, 15] attempted to solve the problem of deformable registration via hierarchical subdivision. However, these methods can only be applied for monomodality registration, and local deformations are linear and small. To the best of our knowledge, no automatic method for registration is perfect, and no generic automatic algorithm has shown to work properly for clinical applications due to the fact that the deformation field is often complex and cannot be estimated well by current automatic deformable registration methods.

The aim of this study is to refine the deformable image registration by manual revision for clinical applications. The B-spline is a powerful tool for modeling 2D or 3D deformable objects. The proposed method is based on multilevel B-spline to interactively revise the misregistration regions by manipulating an underlying mesh of control points in the overlapping region between the reference image and the transformed image in RGB color model. This paper is organized as follows.  Section 2 describes material and proposed registration refinement technique. In Section 3, we show the experimental results on clinical images.  Section 4 concludes this paper.

## 2. Materials and Methods

### 2.1. The Framework of Interactive Multigrid Refinement Algorithm

**Figure 1:**
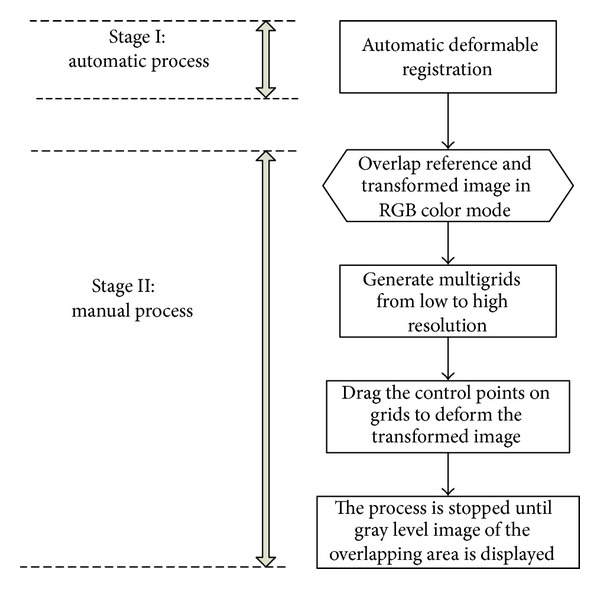
The framework of interactive multigrid refinement algorithm.

We explored digital B-splines to devise an interactive multigrid refinement that consists of automatic process and manual process to improve the accuracy of deformable registration. As shown in Figure 1, the proposed framework of multigrid refinement algorithm consists of two steps. The first is the automatic process in which conventional automatic deformation registration methods or rigid and linear transformation model can be used to coarsely register deformable images. The second is the manual process in which multilevel B-splines are used in the overlapping region of the transformed image and the reference image in RGB model for manual revision. The misregistered areas are represented by colors and the registered areas by gray level to show alignment of the two images. If the automatic registration methods can register deformable images well, there is no need to use the second manual process. In clinical applications, however, the automatic methods often do not register well. The second step will attempt to eliminate the errors visually by manual revision in the misregistered areas. In order to describe the deformation field, we chose the B-splines to model 2D and 3D deformations. Due to the fact that misregistered areas may be large or small in different clinical cases, multilevel B-spline is designed to generate control point mesh at decreasing spacing in a coarse-to-fine manner. Misregistered areas will be reduced coarsely by dragging control points with large spacing mesh. As the misregistered areas are reduced, fine control point mesh will be generated with small spacing. Only control points in misregistered areas need to be revised in the fine level. The process will be stopped until visually satisfying registration results are displayed. Registration of the revised transformed image and the reference image will make the overlapping image in RGB model become gray. We will illustrate the proposed technique in the next sections in detail.

### 2.2. B-Splines and Local Deformation Model

As introduced in the previous section, the goal of interactive multigrid refinement is to reduce the local registration error of deformable registration methods. The nature of local deformation of anatomic structures can vary significantly across patients and ages. Therefore, it is difficult to describe the local deformation via parametric transformations, such as rigid transformation, or affine transformation, which can capture only the global motion of organs. Free-form deformation based on the B-splines is a powerful tool for modeling 3D deformation objects. However, optimization of a cost function associated with the global transformation parameters and the local transformation parameters in the framework of free-form deformation uses an iterative multiresolution search strategy, which is often computationally expensive and prone to local minimum. Generally, the deformation results of free-form deformation contain errors which are visually distinctive from corresponding difference images. Such cases also exist in other kinds of automatic deformation registration methods. To this end, we propose a manual revision process to refine the local deformation model based on multilevel B-splines. Only the misregistration areas are revised by manipulating an underlying mesh of control points. The revision process can be fast and efficient.

To define a local deformation model, B-splines are used for modeling the deformation fields. The domain of the image volume is denoted as  *Ω* = {(*x*, *y*, *z*) | 0 ⩽ *x* < *X*,  0 ⩽ *y* < *Y*,  0 ⩽ *z* < *Z*}. The control lattice Φ is denoted by a mesh of control points Φ_*i*,*j*,*k*_ with uniform spacing overlaid on the domain  *Ω*. Let Φ_*i*,*j*,*k*_ be the *ijk*th control point on the lattice  Φ for *i* = −1,0,…, *X* + 1, *j* = −1,0,…, *Y* + 1, and *k* = −1,0,…, *Z* + 1. The deformation function *T* is defined in terms of these control points by

(1) $$\begin{array}{c} T\left(x,y,z\right)=\overset{3}{\underset{l=0}{\sum }}\overset{3}{\underset{\;m=0\;}{\sum }}\overset{3}{\underset{n=0}{\sum }}B_{l}\left(u\right)B_{m}\left(v\right)B_{n}\left(w\right)\Phi_{\left(i+l)(j+m)(k+n\right)}, \end{array}$$

where *i* = ⌊*x*⌋ − 1, *j* = ⌊*y*⌋ − 1, *k* = ⌊*z*⌋ − 1, *u* = *x* − ⌊*x*⌋, *v* = *y* − ⌊*y*⌋, *w* = *z* − ⌊*z*⌋, and ⌊·⌋ is the lower bound operator. *B*_*l*_, *B*_*m*_, and *B*_*n*_ are uniform basis functions of cubic B-spline defined as

(2.2) $$\begin{array}{c} B_{0}\left(u\right)=\frac{{\left(1-u\right)}^{3}}{6},\\ B_{1}\left(u\right)=\frac{\left(3u^{3}-6u^{2}+4\right)}{6},\\ B_{2}\left(u\right)=\frac{\left(-3u^{3}+3u^{2}+3u+1\right)}{6},\\ B_{3}\left(u\right)=\frac{u^{3}}{6}, \end{array}$$

where 0 ≤ *u* ≤ 1. In general, the transformations that result from cubic B-splines are smooth and able to describe local deformation with few degrees of freedom. In contrast to thin-plate spline [12] or elastic body splines [16], B-splines are locally controlled; in particular, the basis functions of cubic B-splines have a limited support that changing control point Φ_*i*,*j*,*k*_ affects the transformation only in the local neighborhood of that control point. If any data points are added, removed, or modified, B-splines can be computationally efficient. This is the reason we chose B-spines for local deformation model.

The control points Φ_*i*,*j*,*k*_ are the parameters of the B-splines, and the degree of deformation field is essentially dependent on the spacing of control points. A large spacing of control points allows modeling of global deformation with large displacement, and hence, one control point will influence the deformation of large local areas, while a small spacing of control points allows modeling of local deformation within small areas. The resolution of control point mesh generally determines the degrees of freedom. Therefore, hierarchical B-spline refinement can be used to refine the deformation field. We have designed a hierarchical multiresolution B-spline refinement tool in which the resolution of control mesh is increased to revise the deformation field in a coarse-to-fine manner. Let Φ^1^, Φ^2^,…,Φ^*L*^  denote hierarchical control point meshes at different spacings for deformation revision. For each control point mesh Φ^*i*^ and its associated B-spline define a local transformation  *T*_local_^*i*^, and their sum defines the overall local transformation of deformation revision *T*_local_ as

(3) $$\begin{array}{c} T_{\text{local}}\left(x,y,z\right)=\overset{L}{\underset{i=1}{\sum }}{\;T}_{\text{local}}^{i}\left(x,y,z\right). \end{array}$$

In this way, the overall local transformation of deformation revision is represented as a combination of B-splines at increasing resolution of control point mesh. For those misregistered areas, large spacing of control point mesh is generated when misregistered areas are large. After manual revision with related control points, the overall misregistered areas will be reduced. In order to refine the results further, the control point mesh is progressively refined. In this case, the control point mesh at level *i* is refined by inserting new control points to create the control point mesh at level *i* + 1. Therefore, the control point spacing is halved at every step. With the revision of control point at different levels, the final deformation field will be generated to make the reference image coincide with the transformed image.

### 2.3. Manual Refinement in RGB Model

In order to observe the misregistered area between the reference image and the transformed image well, we designed the RGB model. Without loss of generality, we take the case of 2D image registration for explanation. In RGB model, the reference image is shown in the green band, and the transformed test image is shown in the red and blue bands of a color image. Therefore, when the images register perfectly, all three color bands at a pixel will have the same or similar values, producing gray scale. In areas where the images do not register well, the pixels will appear green or purple. Also appearing in green or purple are occluded areas. Thus, the misregistered areas will be displayed in green or purple in RGB model. The manual refinement which is based on the B-splines will revise the deformation transformation through manipulating control points in the transformed image. With the revision of the transformed image, the misregistered areas would be reduced or even eliminated because the local anatomic structures in the transformed images are revised to be aligned with the corresponding structures in the reference image. Meanwhile, the pixels in the misregistered areas that appear green or purple will become gray due to overlapping of the corresponding anatomic structures. The process of manual revision will be stopped until satisfying results are achieved. That is, the overlapping area in RGB model appears gray.

Due to the local geometric differences from large deformation or gray level change between the reference image and the test image, it is not easy to obtain perfect registration between corresponding structures. Therefore, the finally obtained overlapping area does not appear to be gray everywhere. In such case, we take the distinctive edge or salient object as the criterion for manual refinement. For example, if a distinctive edge in the transformed image is revised to be coincided with the corresponding edge in the reference image in RGB model, we consider that the manual refinement is good in such local areas around the distinctive edge. In our experiments, there is no need to revise every control point because the two images have been registered coarsely by automatic deformation registration methods and the misregistered areas are assumed to be limited. Our proposed manual revision is only used to improve the coarsely obtained registration results if the deformable registration methods do not work well. To our knowledge, the whole process of automatic deformable registration methods may not be satisfactory in clinical applications if these registration methods do not work well or if large registration errors are visible. Our proposed method provides a means to aid the process to be successful and allows the user to drag control points to get a better image alignment. If the automatic registration methods do not work well in clinical applications, the clinicians can use our tool to efficiently revise the former registered results directly.

**Figure 2:**
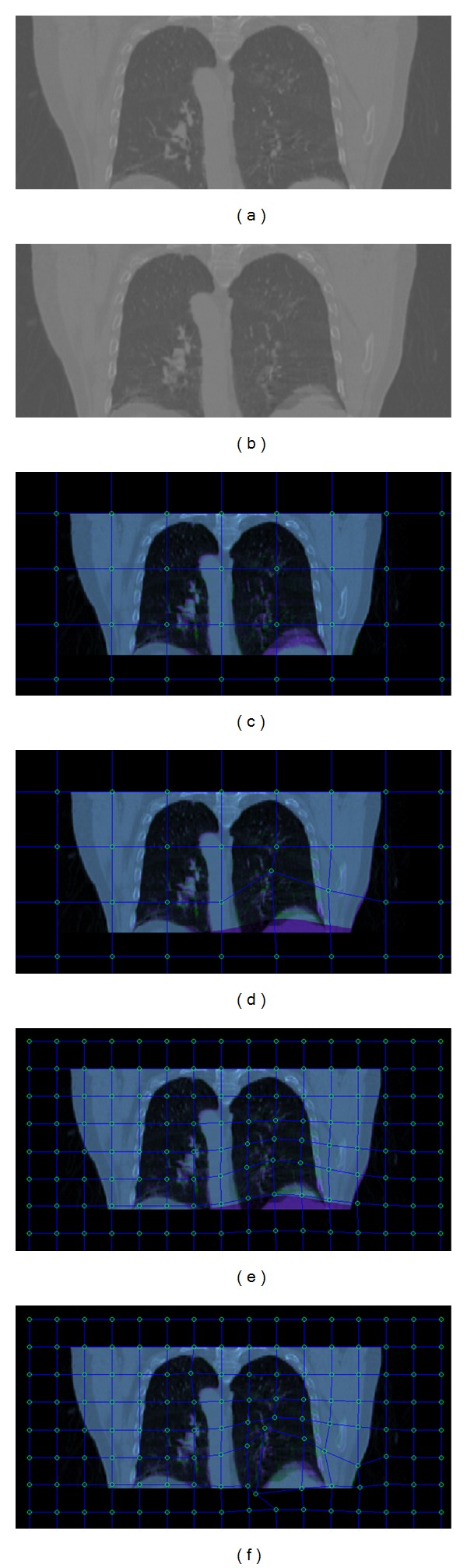
Manual refinement in RGB model. (a) Test image, (b) reference image, (c) overlapping of test image and reference image and large grid mesh in RGB model, (d) manually revise several related control points to reduce the misregistered area (purple or green), (e) progressively refine control point mesh and (f) manually revise related control points further.

To demonstrate the scheme of the manual refinement in RGB model, lung CT images in different respiratory phases are used for illustration. Precise targeting of lung tumors is of great importance in conformal radiotherapy, particularly stereotactic body radiation therapy (SBRT) for lung cancer. The discontinuity of the sliding behavior of the lungs makes the registration of lungs in different respiratory phases very challenging.  Figure 2 shows the lung images in inhale and exhale phases of a patient's 4D CT set. Due to the local deformation of the shape of lungs, we can register the two images by our manual revision technique. As shown in Figure 2(c), the RGB model consists of three color components resulting from the test image and the reference image. Purple or green shows the misregistered area between the two images, and the well registered area appears to be gray. It should be noted that the two input images can be either two unregistered images or two images that have been previously registered, while one is the reference image and the other is the transformed image. That is to say, the proposed technique can be a manual registration tool, or it can be a revision tool to improve the registration results of other deformable registration methods by the clinician. In the control point mesh with large spacing, the mouse dragging of one control point will vary the local deformation of large areas as shown in Figure 2(d). With the decreasing of grid spacing, the revision of two or three control points will deform the transformed image locally and makes it align well with the reference image. The mesh grids can be chosen by clinicians' selection. If large misregistered areas exist in the RGB model, mesh grids with large spacing will be generated. Subsequently, the control point mesh will be progressively refined for further revision until satisfactory results are achieved.

## 3. Results and Discussion

### 3.1. Evaluation Measure

To assess the quality of the registration in images, we have calculated the mean and variance of the squared sum of intensity differences (SSD) [9]. In images before and after deformable registration and manual refinement, the SSD provides an indirect measure of the registration quality as the position of tissue changes. Since the deformation is often local between images, we have manually defined regions of interest (ROIs) around each image and then registered both ROIs independently:

(4) $$\begin{array}{c} \text{SSD}=\frac{1}{n}\sqrt{\sum {\left(I\left(t_{0}\right)-T\left(I\left(t\right)\right)\right)}^{2}}, \end{array}$$

where *I*(*t*_0_) and *I*(*t*) denote the intensities of the images before and after motion and the summation includes all voxels within the overlap of both images. In addition to using the color model to represent misregistered areas visually, we calculate the SSD of the transformed image and the reference image at the same time when each step of manual revision is done. Thus, the evaluation measure SSD can supervise our interactive revision process in realtime. If the value of SSD becomes larger when the clinician is revising the control point, the moving direction of the control point should be inverted to make SSD become small. Meanwhile, the color of local areas in our RGB model is also the indication of alignment. If the local areas of misregistered become small, it is possible to reduce the stepsize to a finer grid for further refinement. The color of local areas becomes gray if registered well.

There are several reasons that we choose the evaluation metric SSD. Firstly, SSD is a good metric to evaluate the difference between two images. If two images registered well, SSD should be small. On the other hand, the evaluation metric of SSD is simple and can be calculated very fast. In our work, the value of evaluation metric should be displayed in realtime when the user is revising the local deformation. That is to say, the evaluation metric should supervise the interactive revision process. Therefore, we choose SSD for the interactive process. Other evaluation metrics, such as normalized correlation and mutual information, are very common for deformable image registration. If the interactive refinement is done, the transformed image and the reference image can be evaluated using kinds of metrics, such as SSD, mutual information, and normalized correlation.

### 3.2. Test Results

**Figure 3:**
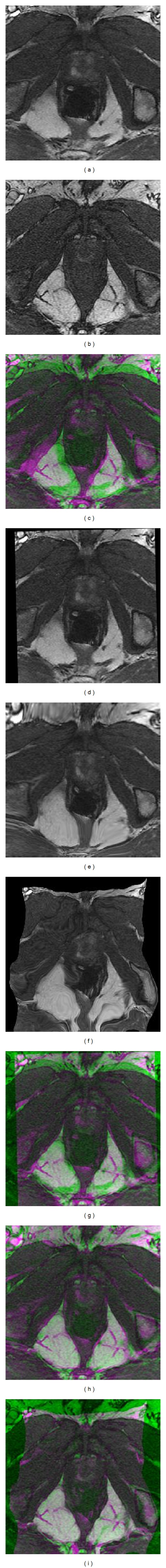
Registration results of affine, FFD, and Demons for two clinical prostate images. (a) Test image, (b) reference image, (c) direct overlapping of (a) and (b) in RGB model, (d) transformed image from affine transformation, (e) transformed image from FFD, (f) transformed image from Demons, (g) overlapping of affine transformed image with the reference image, (h) overlapping of FFD transformed image with the reference image and (i) overlapping of Demons transformed image with the reference image.

Two prostate images of the same patient were acquired from clinical applications. These two images contained both global and local deformations, and the gray intensities were different as well. We chose the affine transformation, FFD, and Demons for coarse registration, respectively. If the two images were registered well, the overlapping area would be in gray, and the color shows the registration errors. As shown in Figure 3, the results of these automatic registration methods all contained errors due to the large local deformable variation between the two prostate images. Affine transformation is a global mapping function for image registration, and there were large registration errors for deformation registration as shown in Figure 3(g). Typically, both FFD and Demons are commonly used deformation registration methods, and their results were much better than those of affine transformation. The color areas in overlapping images as shown in Figures 3(h) and 3(i) were much smaller than the color area in Figure 3(g). The contours of soft tissues were mostly aligned well by FFD and Demons. However, registration errors still existed around some branches of anatomic structures between the two images. The registration results can be improved further by our proposed method. Our proposed tool will generate uniform grids from larger spacing to smaller spacing, and the clinician can select the knot point for mouse draggling. Then, the local area of transformed image will be tuned with the moving of its close knots. The process will be stopped until the color region is eliminated or distinctive branches are almost aligned between two images by the interactive adjustment.

**Figure 4:**
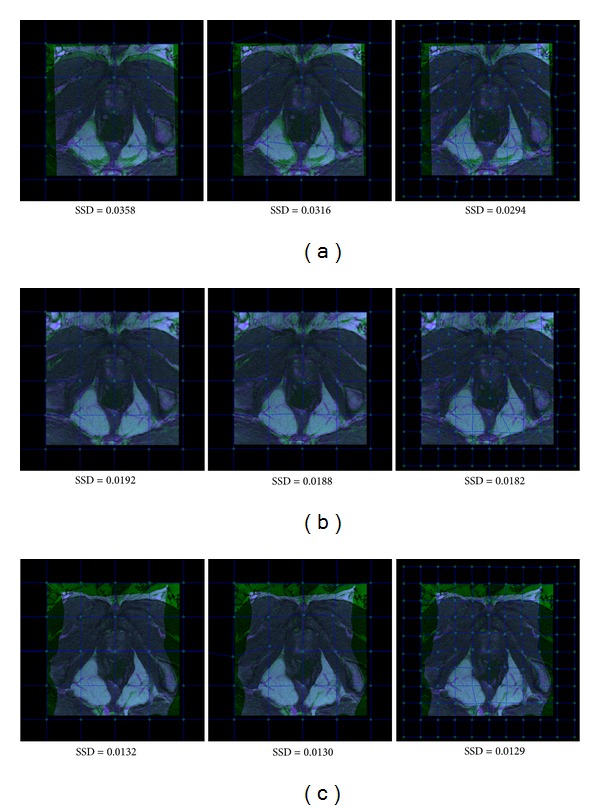
Registration results of manual revision of affine, FFD, and Demons for two clinical prostate images, respectively. (a) Affine, (b) FFD and (c) Demons. The value of SSD shows the performance of registration in overlapping areas. Generally, the lower the SSD, the better the registration results. Note that the SSD for Demons only calculates the areas of overlapping between two images.

The results of manual revision for affine, FFD, and Demons deformable registration methods are shown in Figure 4, respectively. The registration errors of affine, FFD, and Demons were reduced by manipulating progressively refined control point mesh. For affine transformation, our proposed method can improve results distinctively. Typically, the registration results of FFD and Demons were remarkably good due to the high performance of those methods. However, the proposed method can further improve the registration results and reduce registration errors. From the above experimental results, we have shown the effectiveness of the proposed method for deformable image registration in clinical applications. Although our given test data were clinical CT images, other kinds of medical modality can also be used directly. With the development of more complex deformable registration methods, our proposed tool can directly improve the registration results further.

### 3.3. Discussion

In this work, we tried to reduce deformable registration errors in a multigrid process. Large errors are reduced by revising control points in coarse grids, and small errors are reduced by revising control points in fine grids. Generally, our method is similar to multiscale image registration [17, 18], which uses hierarchical multiscale information to recover deformations. We use multigrids to recover deformation from large deformation with sparse grids to local small deformation with fine grids, and those multiscale registration methods are usually using images in different scales (or resolution) for registration, and the deformation can be recovered from global to local. However, focuses are different between our method and those multiscale image registration methods. We concentrate on how to interactively reduce errors of deformable image registration if automatic registration methods cannot work well or registration errors are distinctive in clinical applications. On the other hand, those multiscale image registration methods are trying to register images automatically in a coarse-to-fine manner, and their purposes are generally reducing computational complexity of registration and making the registration more robust and reliable.

The manual revision process may be tedious for clinicians when large misregistered areas exist. That is the truth if the registration error is large and large areas are needed to be revised with more control points. In our work, the framework of the proposed interactive multigrid refinement algorithm consists of two steps: one is the automatic deformable registration method and the second is the manual revision process. We first of all assume that the automatic registration can obtain registration results which are not too bad. That is, the automatic deformation registration can be accurate and reliable to make the misregistered area small in clinical applications. Basically, some deformation registration methods, such as feature-based or intensity-based methods, can generate good results to some extent. Unfortunately, some areas may contain errors practically. Therefore, regions that need to be revised are often limited or very small. Hence, we provide the tool to quickly revise such errors in our proposed RGB model.

In this work, the smoothness is not considered as a metric in the interactive process which may be the limitation of our method. In general, the local deformation of the anatomic structures should be characterized by a smooth transformation [9]. It is known that SSD is a “similarity” measure, which is not the only consideration in evaluating the performance of registration. In some registration algorithms [9, 13], “smoothness” is also considered for the mapping function, but this is not included in SSD. In our interactive process, we consider how to revise the registration error conceptually from multi-grid refinement to make images align better under the evaluation metrics of SSD and color model. B-spline is smooth to some extent to make sure that the deformation field appears to be smooth. However, the “smoothness” is not easy to be considered much in the interactive process. Maybe we can add another metric of smoothness in the interactive process, like (5) in [9]. However, the balance between the similarity and smoothness may be also a problem. Therefore, the finally revised transformed image may be accurate in similarity but not much smooth by our proposed method due to the only use of SSD.

In addition, the evaluation metric of SSD is used for monomodality images. Actually, correspondent features in monomodality images are distinctive for manual revision perceptually. Therefore, the proposed method can obtain reliable and accurate results for monomodality image registration, which is suitable for monomodality images. However, it may not be good and convenient for images in different modalities because of few visual corresponding anatomic structures. If gray scale and image contrast are very different in multimodality images, the manual revision for further refinement of automatic deformable image registration will be difficult. This is also the limitation of our proposed method. In the future, we will also consider the metric of normalized mutual information for multimodality images in our proposed framework.

## 4. Conclusions

In this paper, we propose an interactive tool for deformable registration revision by using multigrid B-spline refinement. This technique can be used to improve registration results of other deformable image registration methods. Experimental results showed that this tool could be used to model deformation accurately and efficiently. The application of this tool can be for medical image registration in clinical cases, such as treatment planning. We believe that it will be a useful tool for clinical applications. In the future work, we will try to register multimodality images well using our proposed method by extracting salient features in the transformed image and the reference image to facilitate visualization.

## References

[1] Peters TM, Cleary KR (2008). *Image-Guided Interventions: Technology and Applications*.

[2] Xing L, Thorndyke B, Schreibmann E (2006). Overview of image-guided radiation therapy. *Medical Dosimetry*.

[3] Xie Y, Chao M, Xing L (2009). Tissue feature-based and segmented deformable image registration for improved modeling of shear movement of lungs. *International Journal of Radiation Oncology Biology Physics*.

[4] Xie Y, Chao M, Xiong G (2011). Deformable image registration of liver with consideration of lung sliding motion. *Medical Physics*.

[5] Hardcastle N, Tomé WA, Cannon DM (2012). A multi-institution evaluation of deformable image registration algorithms for automatic organ delineation in adaptive head and neck radiotherapy. *Radiation Oncology*.

[6] Sotiras A, Davatazikos C, Paragios N (2012). Deformable Image registration: a survey. *Technical Report*.

[7] Pszczolkowski S, Pizarro L, Guerrero R, Rueckert D Nonrigid free-form registration using landmark-based statistic deformation model.

[8] Shusharina N, Sharp G (2012). Image registration using radial basis functions with adaptive radius. *Medical Physics*.

[9] Rueckert D, Sondoda KI, Hayes C, Hill DLG, Leach MO, Hawkes DJ (1999). Nonrigid registration using free-form deformations: application to breast mr images. *IEEE Transactions on Medical Imaging*.

[10] Pennec X, Cachier P, Ayache N Understanding the “Demon's Algorithm”: 3D non-rigid registration by Gradient Descent.

[11] Cahill ND, Noble JA, Hawkes DJ A Demons algorithm for image registration with locally adaptive regularization.

[12] Bookstein FL (1992). Principal warps: thin-plate splines and the decomposition of deformations. *IEEE Transactions on Pattern Analysis and Machine Intelligence*.

[13] Zagorchev L, Goshtasby A (2006). A comparative study of transformation functions for nonrigid image registration. *IEEE Transactions on Image Processing*.

[14] Andronache A, von Siebenthal M, Székely G, Cattin P (2008). Non-rigid registration of multi-modal images using both mutual information and cross-correlation. *Medical Image Analysis*.

[15] Buerger C, Schaeffter T, King AP (2011). Hierarchical adaptive local affine registration for fast and robust respiratory motion estimation. *Medical Image Analysis*.

[16] Ashburner J (2007). A fast diffeomorphic image registration algorithm. *NeuroImage*.

[17] Paquin D, Levy D, Schreibmann E, Lei X (2006). Multiscale image registration. *Mathematical Biosciences and Engineering*.

[18] Thévenaz P, Unser M (2000). Optimization of mutual information for multiresolution image registration. *IEEE Transactions on Image Processing*.

